# Acceptability of financial incentives for breastfeeding: thematic analysis of readers’ comments to UK online news reports

**DOI:** 10.1186/s12884-015-0549-5

**Published:** 2015-05-16

**Authors:** Emma L Giles, Matthew Holmes, Elaine McColl, Falko F Sniehotta, Jean M Adams

**Affiliations:** 1 Health and Social Care Institute, Teesside University, Middlesbrough, North Yorkshire TS1 3BA UK; 2 Institute of Health & Society, Newcastle Clinical Trials Unit, The Medical School, Newcastle University, 4th Floor, William Leech Building, Framlington Place, NE2 4HH Newcastle upon Tyne, Tyne and Wear UK; 3 The Medical School, Newcastle University, Framlington Place, NE2 4HH Newcastle upon Tyne, Tyne and Wear UK; 4 Centre for Diet and Activity Research, MRC Epidemiology Unit, University of Cambridge School of Clinical Medicine, Level 3 Institute of Metabolic Science, Addenbrooke’s Treatment Centre, Cambridge Biomedical Campus, Cambridge, CB2 0SL UK

**Keywords:** Thematic analysis, Netnography, Financial incentives, Breastfeeding, Acceptability

## Abstract

**Background:**

Whilst it is recommended that babies are breastfed exclusively for the first six months, many mothers do not maintain breastfeeding for this length of time. Previous research confirms that women and midwives value financial incentives for breastfeeding, but limited research has explored the wider acceptability of these interventions to the general public. This paper examines opinion towards financial incentives for breastfeeding using reader responses to UK on-line media coverage of a study undertaken in this area.

**Methods:**

This study used netnography to undertake a thematic analysis of 3,373 reader comments posted in response to thirteen articles, published in November 2013, which reported findings from a feasibility study of financial incentives for breastfeeding. All articles were published on one of six UK news websites that achieved a monthly audience of at least five million viewers across laptop and desktop computers and mobile devices during April-May 2013.

**Results:**

Nine analytical themes were identified, with a majority view that financial incentives for breastfeeding are unacceptable. These themes cover a range of opinions: from negligent parents unable to take responsibility for their own actions; through to psychologically vulnerable members of society who should be protected from coercion and manipulation; to capable and responsible women who can, and should be allowed to, make their own decisions. Many views focused on the immediate costs of the intervention, concluding that this was something that was currently unaffordable to fund (e.g. by the NHS). Others contrasted the value of the incentive against other ‘costs’ of breastfeeding. There was some consideration of the issue of cost-effectiveness and cost-saving, where the potential future benefit from initial investment was identified. Many commenters identified that financial incentives do not address the many structural and cultural barriers to breastfeeding.

**Conclusions:**

Overall, those commenting on the on-line UK news articles viewed financial incentives for breastfeeding as unacceptable and that alternative, structural, interventions were likely to be more effective. Further consideration of how best to conduct internet-based qualitative research to elicit opinion towards public health issues is required.

## Background

Breastfeeding promotes the health of babies and infants, with immunological, bonding and nutritional benefits [1]. The World Health Organisation and the UK (United Kingdom) Department of Health recommends that infants are breastfed exclusively for the first six months [2, 3]. Whilst breastfeeding initiation rates in the UK are relatively high, continuation rates fall rapidly. In 2010, 81 % of UK mothers breastfed at birth, 69 % at week one, 55 % at week six, and only 34 % at six months [4]. Thus, efforts to increase breastfeeding initiation and maintenance are promoted throughout the UK [2, 5–7].

Reasons for low breastfeeding initiation and continuation rates vary, and include a lack of support, negative experiences and personal preferences [1]. Other structural and environmental factors impact on breastfeeding rates, such as a lack of information on, and support during, breastfeeding [8], negative opinions of others when breastfeeding in public [9], a lack of available facilities to breastfeed outside the home, and aggressive marketing of artificial baby food [10]. Whilst the benefits of breastfeeding include lower rates of obesity in children and the mother, and a reduction in postpartum depression, costs include difficulty in returning to paid employment, and reduced opportunities for baby-father bonding [11].

One method that has been used to encourage breastfeeding is the use of financial incentives. Although financial incentives have been used to promote a range of other health-related behaviours [12] in developed countries, their use in breastfeeding is a relatively new area, with research to date predominantly identifying only gift-giving and prizes [1, 13]. Conditional cash transfers are more widespread in developing countries (e.g. Latin America) where they have been used to improve a range of health and education outcomes (although not breastfeeding directly) [14]. Although there is a limited evidence base on effectiveness of financial incentives for breastfeeding in developed countries, with no published evidence on randomised-controlled trials; available evidence suggests that incentives (including financial incentives) to promote breastfeeding can be effective [1, 13, 15–19] and are welcomed by mothers as part of a suite of interventions [20].

Research on the acceptability of financial incentives for health behaviours in general, suggests that if health promoting financial incentives (HPFI) are found to be effective and cost-effective, result in benefits for recipients and wider society, and are designed and delivered in particular ways (e.g. using shopping vouchers rather than cash incentives), then they are more likely to be considered acceptable [21]. A particular area of concern is the possibility of recipients ‘gaming the system’, whereby recipients deceitfully profess to be undertaking the behaviour to attain the reward [22].

However, much information on the acceptability of HPFI comes from scholarly writing, rather than primary empirical studies [21]. Where there is empirical evidence, much of this is quantitative in nature [15–19, 23–26]. Furthermore, limited research has been conducted on the acceptability of financial incentives for breastfeeding. Whelan et al. [27] undertook interviews with disadvantaged mothers in England, and with healthcare staff in infant feeding roles, who took part in a pilot incentive scheme. Their findings suggest that financial incentives were viewed positively in terms of providing encouragement for breastfeeding. Nonetheless, ethical concerns were raised, including views that the financial incentives constituted a form of bribery. As far as we are aware, limited previous research has investigated the viewpoints of a more general public towards financial incentives for breastfeeding – rather than mothers and healthcare professionals who had particular experience of these. This is important to ascertain because in a country such as the UK, with a universal health-care system, any breastfeeding related HPFI would be funded by the public purse through taxation. Consequently, any policy decision to implement HPFI is likely to be strongly influenced by perceptions of public opinion.

There is a growing recognition that internet and digital media can have a key role in capturing opinion towards public health issues and interventions [28]. In order to assess the acceptability of financial incentives for breastfeeding in members of the wider public, we examined reader comments posted online in response to a UK news story on incentives for breastfeeding. The news coverage reported on a feasibility study where £200 in shopping vouchers were given to low-income mothers, once they had breastfed for six months [27, 29]. Researchers leading the study issued a press release at the start of the study. This resulted in wide media attention and stimulated debate throughout online news communities [30–39]. Thematic analysis of this news coverage allowed a timely, quick and resource-efficient method to explore attitudes towards financial incentives for breastfeeding. As many types of evidence influences policy makers [40], this information is likely to help contribute to development of a rounded picture of responses to financial incentives for breastfeeding in particular, and health behaviours in general.

## Methods

### Netnographic methods

A variety of methods have been used to understand and analyse information from online sources [41]. One such approach is ‘netnography’. Initially developed for use in the marketing sector to assess consumer attitudes towards products and services [42–44], netnography has now been used more widely, including in the public health field [45].

Netnography is an emerging method of obtaining qualitative data, which enables researchers to gain a naturalistic and immersive insight into online interactions [42–44]. The process of netnography involves sourcing data, using qualitative analysis research software to investigate collected information and forming themes from the data. When conducting a netnography a hypothetical continuum can be argued, ranging from a full ethnographic enquiry using internet sources, to internet-based data analysis [46]. Previous studies – as with our methodological approach – have fallen at the latter end of this continuum and analysed user comments posted on websites and other forums [28].

By using netnography in this study, we were able to gain an insight into opinions surrounding financial incentives for breastfeeding. Limited previous research has thematically analysed reader comments posted online in response to news media stories to explore opinion towards public health interventions. One notable example is a content analysis of public responses to Australian news coverage of tobacco legislation [47].

### News articles of interest

We included reader responses to articles reporting on the ‘financial incentives for breastfeeding pilot study’ published on popular UK news media websites [48] that produced original content (rather than aggregating content from other sources). Popular websites were defined as those that achieved an average monthly audience of at least five million unique viewers per month across laptop computers, desktop computers and mobile devices in April and May 2013 (the most up-to-date usage statistics available at the point when the study commenced). These sites were: bbc.co.uk; guardian.co.uk; dailymail.co.uk; telegraph.co.uk; independent.co.uk; and thesun.co.uk [49]. The first of these is the website of a publicly funded television and radio broadcasting company, whilst the remainder are websites of national daily newspapers (see Table 1 for readership characteristics).

**Table 1:** Readership characteristics

	Website readership adults 15+ April and May 2013 (000 s)	Readership characteristics		
		Age (years)	Gender split	Socio-economic classification
BBC	Information unavailable	Average age 43	Male	Unknown
Daily Mail	8595	Aged 35+	Even gender split	Unknown
Guardian	8301	Range 15-44	Even gender split	ABC1^1^
Independent	4076	Aged less than 45	Male majority	ABC1
Sun	1662	Aged 35+	Male majority	ABC1
Telegraph	7506	Average age 43	Male majority	ABC1

^1^ The National Readership Survey uses social grade as a way to classify individuals. ABC1 individuals are those in higher (‘A’ – upper middle class), intermediate (‘B’ – middle class) or supervisory/junior (‘C1’ – lower middle class) managerial, administrative, or professional occupations [90]

Thirteen articles met the criteria for inclusion: six original news articles, six editorial pieces and one follow-up article reporting ministerial response to the incentive programme (see Table 2 for details on the content and nature of included articles).

**Table 2:** Characteristics of included articles

Source	URL	Format	Title	Reader comments, n	Article stance
BBC	http://www.bbc.co.uk/news/health-24900650	Article	“Breastfeeding mothers offered £200 in shop vouchers”	1121	Mixed
BBC	http://www.bbc.co.uk/news/health-24908678	Article	“Breastfeeding vouchers: Midwives and health visitors verify claims”	0	Mixed
Daily Mail	http://www.dailymail.co.uk/news/article-2501812/Mothers-200-incentive-breastfeed--Poundstretcher-vouchers-Critics-claim-scheme-form-bribery.html	Article	“Mothers to get a £200 incentive to breastfeed…in Poundstretcher vouchers: critics claim scheme is form of bribery"	759	Mixed
Daily Mail	http://www.dailymail.co.uk/news/article-2507269/New-mothers-NOT-paid-breastfeed-Nick-Clegg-says-insists-controversial-voucher-scheme-government-policy.html	Follow-Up Article	“New mothers will not be paid to breastfeed, Nick Clegg says as he insists controversial voucher scheme is not government policy!”	58	Mixed
Guardian	http://www.theguardian.com/lifeandstyle/2013/nov/12/researchers-offer-shopping-vouchers-breastfeed#start-of-comments	Article	“Researchers to offer shopping vouchers to mothers who breastfeed”	330	Mixed
Guardian	http://www.theguardian.com/commentisfree/2013/nov/12/worst-breastfeeding-initiative-shopping-vouchers?INTCMP=ILCNETTXT3487	Editorial	“The worst breastfeeding initiative I’ve ever come across”	546	Negative
Guardian	http://www.theguardian.com/commentisfree/2013/nov/20/not-ashamed-giving-mothers-incentives-breastfeed	Editorial	“No, we’re not ashamed about giving mothers financial incentives to breastfeed”	140	Positive
Independent	http://www.independent.co.uk/life-style/health-and-families/health-news/the-nanny-state-mothers-could-be-paid-to-breastfeed-their-babies-8933503.html	Article	“The nanny state? Mothers could be given shopping vouchers for breastfeeding their babies”	0	Mixed
Independent	http://www.independent.co.uk/voices/comment/vouchers-for-mothers-who-breastfeed-isnt-there-enough-breastmongering-in-the-world-already-8934923.html	Editorial	“Vouchers for mothers who breastfeed: isn’t there enough breast-mongering in the world already?”	9	Negative
Independent	http://www.independent.co.uk/voices/comment/offering-poorer-mothers-200-to-breastfeed-is-barmy-middleclass-lactivism-8935416.html	Editorial	“Offering poorer mothers £200 to breastfeed is barmy, middle-class lactivism”	97	Negative
Sun	http://www.thesun.co.uk/sol/homepage/woman/5259728/Sun-Agony-Aunt-Vouchers-for-breastfeeding-are-an-insult-to-mothers.html	Editorial	“Vouchers for breastfeeding are an insult to mums fund health visitors not token gestures”	3	Negative
Telegraph	http://www.telegraph.co.uk/health/healthnews/10442290/New-mothers-bribed-to-breastfeed-by-NHS-with-200-shopping-vouchers.html	Article	“New mothers ‘bribed to breastfeed’ by NHS with £200 shopping vouchers”	268	Mixed
Telegraph	http://www.telegraph.co.uk/health/10443233/Mothers-might-not-breastfeed-after-taking-200-NHS-bribe-MP-warns.html	Editorial	“Mothers might not breastfeed after taking £200 NHS bribe, MP warns”	42	Mixed

### Reader comments

All included news articles had a facility for readers to leave comments on articles in a semi-anonymous format (e.g. a unique login or pseudonym rather than an individual’s full name) [50, 51]. Although there are certain ‘House Rules’ (e.g. no defamatory comments) that contributors must subscribe to, comments are largely unrestricted and are usually only removed if they feature explicit or offensive material [52, 53]. Most comment sections require users to create a unique login to be able to comment. Users without a login are able to read comments but not comment themselves. In 2013, 73 % of the UK population of just over 64 million accessed the Internet each day [54]. However, given the nature of the internet, readership of the included articles may not have been limited to a UK audience. We were not able to access information on socio-demographic characteristics of the readership of included articles, or of those who left comments and so could not determine the generalizability of commenters.

All comments followed a similar linear format, whereby reader comments were found at the end of the news articles. On all sites, some commenters posted numerous times, thus allowing ‘conversations’ between commenters to develop. Thus, not all comments were made by unique individuals. Additional features on some of the websites included a ‘recommend [the comment]’ function on the Guardian website; a ‘rating [of the comment]’ option on the BBC and Daily Mail websites; and an ‘Editor’s Pick’ section on the BBC website. These elevate comments higher up the thread so that they are more likely to be seen by others.

### Data collection and analysis

All reader comments on included articles were copied and pasted directly from the news websites into a word processing package and then uploaded into NVivo QSR 10 software for storage and analysis.

Inductive thematic analysis was chosen as the most appropriate analytical process. This was because it allowed patterns within the data to be identified, and allowed the data to drive the generation of key themes [55–57]. No a priori theories were chosen to drive the coding process, rather an inductive approach was undertaken, such that theory (codes) were discovered from the data [58]. Following established guidelines [57]; a three-stage process of thematic analysis was undertaken. The first stage involved reading and re-reading individual reader comments, assigning ‘codes’ to relevant and important pieces of information and checking these to ensure no codes had been missed. Once all the codes were generated, the second stage involved merging similar codes, renaming some codes and deleting duplicate codes to create descriptive themes. Lastly, codes were grouped into overarching analytical themes that best described the data [57]. These themes represented the range of public opinions towards HPFI. During the analytical process we analysed the wording of the articles, and in particular whether the stance of the article (positive, negative, or mixed) was likely to have influenced whether the reader comments were positive or negative. We did not find strong evidence that the wording of the articles influenced reader comments, given that comments were a combination of positive and negative responses.

One researcher (ELG) analysed all of the comments, and a second researcher (MH) analysed one third of the comments, with guidance and support from one further researcher (JA). Discussions were undertaken to ensure that coding by researchers was consistent and similar codes and themes were found. Discussions did not identify any major discrepancies arising as a result of the coding process.

The chair of the Newcastle University Faculty of Medical Sciences research ethics committee confirmed that ethical approval was not required for this study. However, we did consider, in-detail, numerous ethical issues arising from this research and report on these in the discussion section.

### Data presentation

The main analytical themes identified by the thematic analysis are presented in the results section. Verbatim quotations are provided to illustrate each theme.

## Results

In total, thirteen news articles were identified and, of these, eleven had reader comments available for analysis (no reader comments were posted on the two remaining articles for reasons unknown). In total 3,373 comments were available and all were analysed. Where financial incentives for breastfeeding were viewed as unacceptable, five main themes were identified: Theme 1) Children are a lifestyle choice; Theme 2) Financial incentives for breastfeeding are discriminatory and divisive; Theme 3) Creating a culture of entitlement; Theme 4) Financial incentives for breastfeeding are personally insulting; and Theme 5) Psychological impact on recipients. Where HPFI were viewed as acceptable, two main themes were identified: Theme 6) Effectiveness and cost-effectiveness; and Theme 7) Generating initial motivation. In addition, two overarching themes permeated the acceptable and unacceptable comments on HPFI: Theme 8) Design and delivery, and Theme 9) Informed choice.

### HPFI viewed as unacceptable

#### Theme 1: Children are a lifestyle choice

There was a view among commenters that having children was a personal, lifestyle choice, and that children were, therefore, their parents’ responsibility. Commenters who noted this emphasised that other people (i.e. taxpayers) should not have to fund an aspect of that choice (i.e. how children are fed). This was linked to an opinion that breastfeeding is ‘free’, compared to bottle-feeding, and so should act as a monetary reward in itself. This theme focused on mothers as ‘others’ and few personal experiences were related.

*“Better still, don’t take it from the taxpayer in the first place. I am getting fed up with, and don’t see why I should fund the lifestyle choice of others to have children.”* [BBC News - © 2013 BBC]

*“Midwives, access to health visitors, ante-natal classes, home births, doctors, nurses all available free on the NHS for advice and support. What more do you want the state to do? Ultimately babies are* [the] *parents’ responsibility.”* [BBC News - © 2013 BBC]

*“If you chose to have children, why should the state,* i.e. *us hard working people, pay for you?”* [Daily Mail - © 2013 Daily Mail]

*“It’s not the government’s job to fork out taxpayers’ money to pay for mothers to feed their own kids. You had the child - you feed it however you like. If it costs money - you pay for it.”* [The Telegraph - © 2013 The Telegraph]

*“Since breastfeeding is free, they’re already better off than those who don’t. Why should I pay my taxes to pay them some more? Having children is a choice. Breastfeeding is a choice. When you make the choice make sure you have the funds to support it - don’t sponge off the rest of us.”* [BBC News - © 2013 BBC]

#### Theme 2: Financial incentives for breastfeeding are discriminatory and divisive

A number of commenters asserted that the incentive scheme was unfair. Many of these comments were from individuals who appeared to be mothers who had been unable to breastfeed themselves or from those who did not have access to an incentive scheme. These commenters felt the scheme would discriminate against those like themselves. Other commenters argued that the scheme was unfair as it discriminated against other, ineligible women, who were not necessarily like themselves and to whom no personal connection was made. In particular, the targeted nature of the scheme to low-income parts of one region was identified as a potential source of social inequality.

*“I find these constant rewards deeply divisive. What about the person in the next area who does not qualify?”* [BBC News - © 2013 BBC]

*“If this ‘benefit’ had been introduced when my children were born and we didn’t receive it, it would’ve been discriminatory.”* [BBC News - © 2013 BBC]

*“I think this is disgraceful. What about those who can’t breastfeed due to medication, not producing enough, illness etc., or if single dads are raising their children. Why should they miss out? It’s a form of discrimination.”* [Daily Mail - © 2013 Daily Mail]

*“And this is why it should not be a paid incentive. It effectively punishes, financially, those who can’t but want to.”* [The Independent - © The Independent]

*“Not all women can breastfeed this is discrimination.”* [The Sun - © The Sun]

*“Giving an incentive is all well and good but sometimes a Mum just cannot breastfeed. My little boy simply wouldn’t suckle and I so wanted to breastfeed him. I went to many different breastfeeding groups for help and nothing made any difference… would I have been entitled to these vouchers* [*?*] *and would it have made a difference - probably not!”* [BBC News - © 2013 BBC]

*“These initiatives also exclude women who may not be able to breastfeed - and there are many women who cannot. Also, what about poor women who are also working in low paid, temporary or zero hour contracts? Pumping breaks are not a legal right in the UK. It seems divisive and inherently unfair to reward women for the way that they feed their children.”* [The Independent - © The Independent]

#### Theme 3: Creating a culture of entitlement

Some commenters believed that the incentive scheme would foster a culture of entitlement. In particular, it was felt that if individuals were rewarded for one behaviour, they would demand payment for other health and pro-social behaviours. Commenters argued that this ‘culture of entitlement’ would erode personal responsibility for health behaviours, with individuals only pursuing health behaviours for financial reward, rather than the health, and other benefits, of breastfeeding.

*“There are already too many parents who don’t do the right thing unless there’s ‘something in it for them’ and this just encourages that same mentality.”* [BBC News - © 2013 BBC]

*“There seems to be a mentality within society that the only way to get people to do things is to reward them for it.”* [BBC News - © 2013 BBC]

*“I have an immediate knee jerk hostility to this study because if proven, the hypothesis would lead to the adoption of an incentive scheme to promote healthy behaviour. This would make a responsibility or positive choice, into a chore that must be rewarded or can be ignored. Even if this offers reasonable short-term value in health improvement, this proposition cannot be continued for ever, and it would be hard to exit. We cannot afford to pay people to act responsibly for themselves, and if we start doing so then we risk encouraging them to give up those responsibilities. Then it will be even harder to make them take these up again in the future. Why spend money researching a solution that carries a significant risk of eventually making things worse?”* [The Guardian - © 2013 The Guardian]

*“It is patronising and creates an expectation of entitlement, as we are not usually paid to do the right things in society.”* [The Guardian - © 2013 The Guardian]

#### Theme 4: Financial incentives for breastfeeding are personally insulting

Commenters, who appeared to be predominantly mothers themselves, often interpreted the incentive scheme as suggesting they were incapable of making the right choice for themselves and their baby. There was a strong feeling that this was personally insulting to mothers. This was linked to issues of the ‘nanny state’ interfering in everyday lives where it was neither wanted, nor needed. This theme reflected a view of women and mothers as capable and responsible individuals.

*“I am finding the new initiative patronising and if I may say unfair. Women in Britain are capable of making the right decisions with regards to breastfeeding.”* [BBC News - © 2013 BBC]

*“I don’t agree with paying people to breastfeed as this project is proposing, I think its patronising to assume people don’t want to do the best for their children, whatever their income, unless you pay them.”* [The Guardian - © 2013 The Guardian]

*“I think I’ve heard it all now. Next the government will pay people not to smoke, pay people to eat fruit rather than fatburgers, pay kids to walk to school rather than get a lift in mummy’s diesel polluter. Has the world gone stark staring mad? Is there no end to this do-gooder, nanny-state nonsense?”* [BBC News - © 2013 BBC]

*“Nanny state literally!”* [BBC News - © 2013 BBC]

#### Theme 5: Emotional impact on recipients

Lastly, many of those who did not support the breastfeeding incentive scheme viewed it as reinforcing a feeling of failure in mothers who were unable to breastfeed, contributing to additional guilt and low mood at a time when mothers were already psychologically vulnerable. The double insult of both being unable to breastfeed and being ineligible for the financial incentive was particularly highlighted, and the incentive scheme was identified as manipulative or coercive. In contrast to some other themes, here there was particular concern for new mothers as fragile individuals who needed protection.

*“There is enough pressure on new mums as it is to breastfeed, which makes new mums feel like they are a failure if they can’t do it.”* [BBC News - © 2013 BBC]

*“I was simply unable to satisfy the demands of my eldest child and he ended up in hospital because he failed to gain weight. I felt like a failure and it was a very emotionally tough time - without added financial pressure”* [BBC News - © 2013 BBC]

*“It’s time to consider that some mums actually CAN’T BF* [breastfeed] *through no fault of their own. It’s heartbreaking for those of us who WANT to but CAN’T but to see other mums rewarded is so wrong.”* [BBC News - © 2013 BBC]

*“Very badly thought out idea, there are many women who are unable to breast feed for different reasons, as well as those who simply choose not to for whatever reason. For those that cannot it is just going to make them feel worse.”* [Daily Mail - © 2013 Daily Mail]

*“Ultimately the decision to breastfeed is up to a mother, there* [are] *enough pressures on new mums and this is just another way to pressurise a new mum at a very vulnerable time.”* [Daily Mail - © 2013 Daily Mail]

### HPFI viewed as acceptable

#### Theme 6: Effectiveness and cost-effectiveness

In contrast to the overwhelming negativity towards the financial incentive scheme, there was a minority view that it was important to find out whether the intervention was both effective and cost-effective. Some commenters stated that it was useful that the pilot scheme was going ahead (despite opposition), because the results of the pilot would be valuable to inform future policy and practice. Some of these comments included detailed knowledge of the nature of public health science.

*“This is a research study funded by the MRC* [Medical Research Council] *who independently select which studies to help fund. This isn’t the government handing out benefits to mums! If research outcome*[s] *in the pilot study are good, it creates discussion as to how take it forward. The government are not just going to say ‘ok, £200 for all mums’. The question being asked is about the efficacy of financial incentives - worth exploring!”* [BBC News - © 2013 BBC]

“It’s a pilot. If successful, a nationwide pilot could be rolled out”. You can’t knock people for trying things. Why don’t we decide whether it is a decent idea *after* the pilot is finished, and the results can be assessed? Don’t bash an idea just because it’s different, that’s kind of the point of ideas…” [BBC News - © 2013 BBC]

*“The point is it is a pilot. If it works - great we have a proven method of improving the health of children. If it does not work, then fine we have learnt not to use this method. Cost very little and not coming out of the tax payers pocket. What is the problem?”* [BBC News - © 2013 BBC]

*“If I’m not mistaken this is a trial run by researchers at a university. Before we all jump to premature conclusions about the results shouldn’t we let them at least gather the evidence? What could possibly be wrong with that, or is that the new standard for science, judge the outcome before the trial?”* [The Guardian - © 2013 The Guardian]

The idea that a financial incentive for breastfeeding could be a social investment was particularly prominent in this theme. Commenters identified that by spending a small amount of money on incentives up-front, substantial future health care and other social costs could be avoided. These arguments focused on the potential for financial incentives for breastfeeding to be not just cost-effective, but cost saving.

*“£200 to improve a child’s immunological and brain function. I’d call it an investment as that £200 potentially reduces the child’s need for medical interventions, antibiotics and may also improve the child’s educational ability leading to improved opportunities. A better investment.”* [BBC News - © 2013 BBC]

*“£200 in vouchers for a breastfed baby is a lot less than the millions it costs the NHS each year to treat babies who are not breastfed!”* [Daily Mail - © 2013 Daily Mail]

*“This should be the NHS’ top priority! It’s cheaper to prevent illnesses by promoting breastfeeding than to treat* [consequences such as] *health problems later.”* [Daily Mail - © 2013 Daily Mail]

*“I think this voucher is a great investment to avoid future healthcare costs.”* [The Guardian - © 2013 The Guardian]

*“I don’t mind some incentives. The health benefits are proven, and there is a long-term saving to the NHS.”* [The Independent - © 2013 The Independent]

*“Whether or not you like the ‘nanny state’, the reality is that you already fund the bad health and lifestyle choices of the population. If a little bribe is needed to save money, what’s wrong with that? Granted, it’s not ideal that it’s necessary, but if so many women aren’t responsible enough to look after the well-being of their children then there is little alternative. You’d be paying more anyway.”* [The Telegraph - © 2013 The Telegraph]

#### Theme 7: Generating initial motivation

In a more positive light, the scheme was viewed by some commenters as a way to encourage mothers to start breastfeeding. After this initial encouragement, the hope was that mothers would get into a routine and continue breastfeeding. On occasions, these comments, as well as those in the previous theme, were depersonalised and tended towards ‘othering’ – if other people (not me) need this small encouragement, then they should have it.

*“The financial incentive is a good idea to get the ball rolling why not?”* [The Guardian - © 2013 The Guardian]

*“I think a financial incentive is a good idea if it attracts the attention of women in social situations where they are less likely to do it - it could make it temporarily more acceptable and great oaks out of acorns grow.”* [The Guardian - © 2013 The Guardian]

### Factors affecting implementation and delivery of incentives

#### Theme 8: Questioning the design, delivery and impact of incentive schemes

The articles generated a great deal of comment around the practicalities of the scheme, the format and value of the incentive itself, and questions around monitoring and funding. Concerns around practical issues arose, such as questioning when women would be provided with the incentive; whether mothers would still be eligible for the incentive if they initiated breastfeeding but subsequently stopped earlier than recommended; how the scheme would distinguish between mothers who chose not to breastfeed and mothers who could not breastfeed; and whether mothers would be eligible for some (or all) of the vouchers if they combination-fed their child. These comments reflected a lack of detail in the news coverage but highlight the complexity of designing such a scheme [59] – and then communicating that the scheme has been thoroughly thought through to the public, as well as potential recipients.

*“At what point will these women be given the vouchers?”* [BBC News - © 2013 BBC]

*“…and what if a genuine mother starts to breastfeed and then changes her mind because of complications? Will she have to pay it back?”* [BBC News - © 2013 BBC]

*“Where is the detail on the criteria for those who choose not to breastfeed and those who cannot breastfeed (for various reasons - baby won’t take to it, milk is insufficient, mother has to stop due to the common intense pain etc.). There must be a can’t/won’t distinction in this and policing that will be impossible. Does a mother who wants to breastfeed but physically can’t, get the £200??”* [BBC News - © 2013 BBC]

*“Do you have to exclusively breastfeed? Won’t most mothers say they are breastfeeding (and maybe supplementing with formula?)”* [Daily Mail - © 2013 Daily Mail]

There was also a view that the incentive offered (£200 in supermarket vouchers) was not large enough to incentivise behaviour change, or compensate for the effort, pain and financial cost (e.g. for nursing clothing and equipment) required for breastfeeding. This contrasts with the view (expressed in Theme 1) that breastfeeding is free and hence without ‘cost’. Commenters also suggested that it may be more appropriate to provide vouchers for child-specific shops than supermarkets, although little rationale for why this would either be more acceptable or more effective was given.

*“A bribe of just over £1 per day* [is] *not exactly an incentive.”* [BBC News - © 2013 BBC]

*“A popular motivational theory suggests that in order to be motivated to do something, one must value the reward and believe that one’s efforts will achieve the rewarded outcome. £200 in shop vouchers isn’t going to be enough of a reward to overcome societal rejection, lack of money for pumps, pads, and nursing bras, no training, and no time to feed or pump.”* [The Guardian - © 2013 The Guardian]

*“Why not make them Mothercare/child-centred vouchers?”* [The Telegraph - © 2013 The Telegraph]

There was also concern over how the financial incentive scheme would be monitored. Comments in this theme reflected a distrust of others who may ‘game the system’, i.e. seek the incentive by claiming to be breastfeeding when they were not. It was considered easy for mothers to say that they were breastfeeding when they were not, and difficult for midwives and health visitors to know otherwise without directly witnessing breastfeeding. These comments were particularly distrustful of mothers.

*“How are they going to stop the cash being claimed fraudulently, go round and check at feeding time?”* [BBC News - © 2013 BBC]

*“And how will they ‘police’ this?”* [BBC News - © 2013 BBC]

*“How is this going to be monitored???? Will we not have hundreds of claimants who will make false claims?”* [BBC News - © 2013 BBC]

*“Cannot prove these Mums are breast feeding, another fix for easy money!”* [Daily Mail - © 2013 Daily Mail]

*“There’s no way of checking who is actually breastfeeding, all you have to do is tick a box.”* [The Telegraph - © 2013 The Telegraph]

Finally, there was considerable focus on how the incentive scheme would be funded – with an assumption that this would, ultimately, be the taxpayer. In contrast to those who suggested that financial incentives for breastfeeding could be cost-saving, comments in this theme referred to the current financial climate of debt, deficit and austerity, suggesting that additional expenses could not be afforded.

*“Where does this £200 come from? Which budget will suffer?”* [BBC News - © 2013 BBC]

*“More wasted money to be funded yet again by the TAXPAYER!!”* [BBC News - © 2013 BBC]

*“What gives the government the right to give tax payers money away like this?”* [BBC News - © 2013 BBC]

*“I thought the country was broke where on earth is the money* [going to] *come from?”* [Daily Mail - © 2013 Daily Mail]

*“Given that our maternity services are in crisis, and there are many disadvantaged families living in poverty without the social, financial and medical support that they need it seems morally indefensible to plough money into a scheme like this.”* [The Independent - © 2013 The Independent]

*“Offering a £200 voucher if you breastfeed your child flies in the face of current Government efforts to save money by cutting costs in support of our society right across the board.”* [The Telegraph - © 2013 The Telegraph]

#### Theme 9: Inequitable impact on personal choice

Commenters indicated that breastfeeding is a mother’s choice and that mothers should not be bullied or bribed into engaging in the behaviour. The incentive scheme was also viewed by some as being inconsiderate, in not taking into account the lifestyles of mothers who may have to return to work, and thus have no choice but to use artificial baby food. Ultimately, the need for infant feeding to be a personal decision without outside influence was viewed as important.

*“It’s a personal choice and no one should be bullied into* [it]*.”* [BBC News - © 2013 BBC]

*“Breastfeeding is not something that should be forced or bribed for, it’s a choice.”* [Daily Mail - © 2013 Daily Mail]

*“As a midwife I find this scheme indefensible. Breastfeeding is a personal choice, women should not be coerced into it with financial incentives. I also find this incredibly discriminatory towards women who are formula feeding.”* [Daily Mail - © 2013 Daily Mail]

*“I also believe that it should be the choice of the mother; and women shouldn’t be made to feel guilty about making whatever suits them and their baby best.”* [The Guardian - © 2013 The Guardian]

*“Then they could make an informed and free choice about how they want to feed.”* [The Independent - © The Independent]

It is also worth mentioning that many commenters suggested that alternative approaches to encourage breastfeeding would be more effective, more acceptable, more relevant and better value for money than financial incentives. We have not reported these results given that they are not directly related to the aim of this research, but it is important to say that education and support; fostering a pro-breastfeeding culture; creating an environment which facilitates breastfeeding; improving maternity services; and providing other necessities for babies and mothers after birth were all suggested as alternative approaches to financial incentives for encouraging breastfeeding.

## Discussion

### Statement of principal findings

This is the first analysis of reader comments posted in response to online news articles covering financial incentives for breastfeeding that we are aware of, and one of only a few to use ‘netnography’ to study a public health topic. It contributes to the small existing empirical evidence base on the acceptability of financial incentives for breastfeeding in particular, and health behaviours in general. In response to the coverage in thirteen articles of a pilot study for financial incentives for breastfeeding on six UK online news media websites, 3,373 comments were posted. Most comments were not supportive of financial incentives for breastfeeding, although there was some support from those who took a pragmatic viewpoint towards both breastfeeding promotion and production of research evidence.

Comments revealed a range of views about new mothers who did not breastfeed: from negligent parents unable to take responsibility for their own actions and choices who could not be trusted; through to psychologically vulnerable members of society who should be protected from coercion and manipulation; to capable and responsible women who can, and should be allowed to, make their own decisions. The stark monetary aspect of financial incentives also raised a range of contrasting opinions. Many focused on the immediate costs of such an intervention, concluding that this was something that was currently unaffordable. Others contrasted the value of the incentive against the ‘costs’ of breastfeeding - coming to a range of different conclusions depending on how these ‘costs’ were considered. Finally, there was some consideration of the issue of cost-effectiveness and cost-saving, where the potential future benefit from initial investment was identified.

Overall, alternative approaches to encourage breastfeeding were felt to be more effective, acceptable, relevant and better value for money than financial incentives. Proposed alternatives focused on providing supportive environments and cultures for breastfeeding and reflect academic literature on the barriers to breastfeeding and what may work to increase breastfeeding rates [60].

### Strengths and limitations

Whilst we were able to download all of the available comments on the thirteen included articles, some comments had been removed by website administrators. Thus, we were unable to view or analyse those comments considered offensive or inappropriate by website managers. However, it is possible that these comments represented legitimate views and their exclusion may lead to some bias in our results. We also considered the readership of websites, the type of publication, the wording of included articles, and whether initial negative comments resulted in other negative comments. We did not find strong qualitative evidence that these factors influenced reader comments, as many comments in each theme were similar regardless of the website on which they were posted. We did not explore this quantitatively.

It is argued that those interested in rigour should be concerned, not with the data collected, but the data collection process [61]. Thus, we had to consider whether the online comments and commenters were representative of the range of views of the public. Our inclusion criteria, focusing on the most popular UK news websites, means that we are likely to have captured some of the most-read news articles on this topic. We also analysed all of the reader comments, to help avoid missing key issues raised by commenters.

A further potential limitation concerns the type of people who commented on the news articles. It is argued that people who read and comment on online news stories may be different to those who do not, in both predictable and unpredictable ways [62]. For example, those who comment may hold stronger views than those who do not; they may be socially similar to each other [61]; and have a particular interest in the topic. Certainly, those who comment appear to represent a minority of readers on any website [63]. All of these issues will limit the representativeness of this sample. Additionally, as we were unaware of the nationality and location of the commenters we are unable to say how representative they are of the British public, or even a worldwide audience. That said, the issue of representativeness is not unique to netnographic results. Given the variety of views expressed both in favour and not in favour of incentives, and the many apparent contradictions in the data, we have confidence that we have captured the range of opinions provided. However, this does not necessarily mean that the sample is wholly representative of the general population, and further research would need to be done to establish whether these opinions are replicated in a representative sample.

It is also possible that the anonymity of the internet results in profession of untruthful views and has meant that some of the comments are not truthful expressions of viewpoints. That said, research has found that individuals may be more inclined to be untruthful face-to-face rather than in an anonymous online space [64]; and given that over 3,000 comments were analysed with significant overlap in opinions, we feel that the full corpus of comments accurately reflect the range of opinion present.

Whilst the methods used may not allow for generalisable findings, given that we are unaware of sample characteristics [65, 66]. it is worth emphasising that there are pockets of opinion that assert that applying the very concept of generalisability to qualitative research is erroneous – particularly if applied in the same way as it would be to quantitative research [66]. Given that our findings overlap with previous research on acceptability of HPFI in general, it could perhaps be concluded that they give an indication of the range of opinion present on the issue [21].

The netnography approach allowed a wide range of opinions to be gathered. This is an inherent strength in that we have been able to analyse a large number of (more than 3000) opinions [44] with few resources [67], in a relatively short space of time [61, 68]. This approach required considerably fewer resources than a traditional interview or focus group approach as we did not have to recruit participants or arrange, conduct and transcribe interviews. It also meant that researcher influence on findings was substantially reduced since there was no researcher present at the time when the comments were posted; although there remains potential for such influence during analysis and interpretation and for the authors of the articles to bias commenters’ opinions (although we did not find evidence of this).

### Interpretation of results

The themes we identified were, on the whole, not unique to financial incentives for breastfeeding, and reflect previous findings around acceptability of HPFI in general [21, 69, 70], as well as financial incentives for breastfeeding in particular [27]. However, our findings extend previous work on the acceptability of financial incentives for breastfeeding to a new group of participants (i.e. not specifically mothers and health professionals only with experience of a financial incentive scheme) and to a wider range of issues than previously studied (i.e. whether incentives should be cash or voucher only) [71, 72].

The strong imbalance of views found, falling heavily against financial incentives for breastfeeding is atypical of current literature on HPFI in general - which tends to be more balanced [69, 73]. It is possible that breastfeeding is perceived to be less personally controllable than, for example, smoking. Thus there is a strong view, often expressed in the context of what appears to be personal experience, that some mothers are unable to breastfeed, through no fault of their own. In this context, becoming ineligible for a financial reward is seen as inappropriate punishment – although it could be possible that online reader comments select for negativity, especially when negative information is provided in the article [74].

There was also a concern that HPFI may create a ‘culture of entitlement’, which has not been previously described. However, there is a literature on the potential for financial incentives to erode intrinsic motivation for behaviour change, with laboratory and community-based evidence of the short-term deleterious effects of incentives on intrinsic motivation [22, 71, 75–78].

Perceived, if not explicitly stated, similarities between breastfeeding and other health-related behaviours were also evident. For example, in the literature on the acceptability of financial incentives for smoking cessation, there is a view that quitting smoking is cost-saving to individuals and that this should be enough in the way of financial reward and incentive in itself [79, 80]. Similarly, we found a belief that breastfeeding was ‘cheaper’ than bottle feeding and hence should be considered financial incentive enough. This has been reported as a perceived benefit of breastfeeding in other contexts [81]. However, other commentators recognised some of the wider, and not just financial, costs and benefits of breastfeeding to mothers, and society, including impacts on maternal employment, and reduced long term healthcare costs for children.

These two issues – of creating a culture of entitlement and saving money by breastfeeding - may indicate a need to position financial incentives for breastfeeding, if they were to be widely adopted, as a temporary added bonus, as opposed to a continued reward for the behaviour that individuals will expect to receive [76]. This may help to mitigate against mothers feeling punished if they are ineligible for the financial incentive; and for other individuals becoming annoyed at mothers receiving a double incentive – saving money by bottle-feeding whilst also receiving a financial reward for breastfeeding. Even in these circumstances it would still be important to ensure objective monitoring and evaluation was undertaken to ensure the scheme is not open to abuse.

Some commenters also suggested that infant feeding represents a personal ‘choice’ that parents should be free to make without undue pressure one way or the other. That a ‘choice’ truly exists is contested by many – including other commenters in the study – and commercial influences on the discourse surrounding the ‘choice’ debate in breastfeeding have been highlighted [82].

Opinions on the use of financial incentives for encouraging breastfeeding were often deeply grounded in individual’s personal experiences. This is not something that is as evident in work on the acceptability of HPFI for other behaviours [69]. This may reflect the reality that the great majority of adults have direct experience of infant feeding in contrast to, for example, smoking cessation. This personal ‘expertise’ is important to take into account when both designing interventions, and describing and ‘selling’ them to the public.

### Ethical considerations

Netnography is still a relatively new approach to data collection and analysis and there is limited guidance on the ethics of using this approach. As such, we feel it is appropriate to explicitly discuss the main ethical issues raised in some detail.

The comments analysed here were not provided for research purposes, and commenters are not aware that we have used them for this. They have, therefore, not provided informed consent to take part in the research. However, comments were posted on publically available websites, and commenters were made aware that others could view their comments [83] (see DailyMail.co.uk). We sought permission from the websites involved to use their content and adhered to copyright guidelines throughout [84]. Discussion with the chair of the Newcastle University Faculty of Medical Sciences Research Ethics Committee confirmed that ethical approval was not required for this research.

We did not identify ourselves as researchers and observers to the online communities. This was primarily because we chose, as others have done [45], not to interfere with comments and discussions as they developed. This meant that as researchers we did not influence the data included in the research – as might have been the case in more traditional interviews or focus groups.

To preserve the anonymity of commenters, we have been careful not to include any details in quotations that could have identified the commenter. We also followed best practice guidance provided by the British Psychological Society [85].

Finally, we have not provided a summary of the results to participants. This was deemed infeasible as many of the commenters are unlikely to view the threads after their initial posts, even if we posted headline results on the individual websites from where comments had been taken. It is an acknowledged limitation of online research that it is much harder to debrief subjects than when using traditional research methods [83]. Overall, in considering the ethics of internet-based research, we have adopted a ‘pragmatic perspective’, recognising the difficulties of conducting ‘covert’ research [46]; but at the same time respecting the privacy and anonymity of ‘participants’.

### Implications for research, policy and practice

The finding that £200 was considered by some commenters to be so large as to be manipulative; but by others, too little recompense for the effort required to breastfeed, highlights the problems of setting the value of HPFI ‘just right’. It also implies that if incentives are found to be effective then further research needs to consider what an acceptable value would be both in terms of effectiveness, acceptability and individuals’ willingness to pay for incentives (via taxation) [26, 86]. Given the finding that alternative approaches to promoting breastfeeding were perceived more positively than financial incentives by commenters, any incentive scheme would have to also provide education. Concerns about ‘gaming the system’ could be allayed by providing information for participants about how incentive schemes are ‘policed’. Information on effectiveness and cost-effectiveness could also be provided.

The deep contrasts and contradictions in the views expressed highlights that a ‘one size fits all’ approach to behaviour change is unlikely to be effective. Tailoring and targeted of a range of different intervention approaches is likely to be more effective and acceptable [87, 88]. Research may need to explore how incentive interventions can be tailored whilst remaining both effective and cost-effective. Given that the targeting of incentives to low-income communities was one aspect identified as particularly unacceptable in this work, further research comparing the acceptability of universal versus targeted financial incentives may be useful.

One of the most common views expressed was that further education and support for breastfeeding should be provided in addition to, or instead of, financial incentives. A lack of awareness of existing educational efforts to promote healthy behaviours has been found in previous research [69]. This highlights that whilst health professionals may recognise education as a first step in the behaviour change toolkit, the public may perceive the need for education to play a bigger role. It may also suggest that existing health education is inaccessible to the public. Greater account of current levels of health literacy may be required [89].

Finally, netnography is a relatively novel methodology in the public health arena. Further research is needed to determine the validity of this approach to eliciting public opinion towards public health issues. Consideration of the value of different netnographic approaches may also be useful. For example, immersing oneself in the online environment – rather than simply downloading comments, as we did in this study. Additionally, research could further explore what proportion of readers of different websites post responses to online news articles and how generalisable this data is. The limited existing research in this area suggests that it is a small proportion of online news readers who comment on news articles [63].

## Conclusions

The evidence from this netnographic study, involving thematic analysis of online reader comments, suggests that financial incentives are largely unacceptable for encouraging breastfeeding amongst this group of individuals. There were concerns that the scheme could not be objectively monitored or adequately funded; that the scheme could be discriminatory and even insulting to mothers who wanted to breastfeed but who were unable to; and that the value of the incentive was insufficient compared to the effort involved in the behaviour. Whilst there were some commenters who viewed financial incentives for breastfeeding as acceptable, this acceptability centred on pragmatic reasons, such that the scheme could be effective and cost-effective in the long run by saving the NHS money; that it provided an initial encouragement to mothers; and that an evidence base was required to inform the debate. Given what is known about the effectiveness of HPFI in general, and mothers’ and midwives’ preferences for financial incentives for breastfeeding in particular, further research is needed to determine how financial incentive interventions can be designed and communicated to the public to maximise acceptability and so achieve their potential for behaviour change. Further consideration of how best to conduct internet-based qualitative research to elicit opinion towards public health issues is also required.
